# Hypothermia in burns intensive care: use of the intravenous temperature management system Thermogard XP^®^

**DOI:** 10.1186/cc11273

**Published:** 2012-06-07

**Authors:** Meyyappan Nachiappan, Dilnath Gurusinghe, Sameer Bhandari

**Affiliations:** 1 Yorkshire Regional Burns Centre, Pinderfields General Hospital, Mid Yorkshire Hospitals NHS Trust, West Yorkshire, UK

## Introduction

The care of the patient with major burns in the ICU is a complex and challenging task. They differ from the other critical care patient groups in several ways. One of the major challenges faced is confronting their hypermetabolic state and temperature management [1]. It is widely known that major burn injury is associated with the most profound of hypermetabolic responses to a pathological state. Hyperthermia that is non-infectious is a feature of the systemic inflammatory response to this. In burns intensive care, the disproportionate increase in metabolic rate to small rises in core temperature can have significant impact on resuscitation and prognosis. The pathophysiology of the hyperthermic response in major burn injury is poorly understood. It could be secondary to an infective etiology or a metabolic response to the systemic inflammation. Irrespective of the reason, sustained hyperthermia above 40°C can culminate in cellular injury and death [2].

The hypermetabolic response starts within the first 5 days of the major burn and can last for a year after the injury. Because of the ongoing systemic inflammatory stimulation, patients with major burns often have pyrexia and their thermoregulatory system reset at a higher baseline temperature around 38.5°C [3].

While therapeutic cooling is widely used in neuro intensive care in the management of hyperthermic brain-injured patients and in patients after out-of-hospital cardiac arrests, there is very scarce literature available on the management of hyperthermia in burns intensive care. We, in this article, would like to share our experience of using the intravascular temperature management system (IVTM) Thermoguard XP^® ^in our unit to manage refractory hyperthermia in patients with major burns. We report the responses of two major burns patients to core intravascular thermoregulation during periods of severe hyperthermia (>40°C).

### Case 1

A 24-year-old male had sustained 80% mixed depth, total body surface area (TBSA) flame burns following a road traffic accident. He had no significant past medical history. Initial resuscitation including endotracheal intubation and fluid resuscitation was instituted in the nearby district general hospital and was transferred over to our burns ICU without much delay. On admission to the ICU, detailed assessment of the burn injuries revealed second-degree and third-degree burns involving the trunk, abdomen, back, upper and lower extremities. Initial temperature recorded was 34°C. He responded to external warming, which included nursing in a warm environment, use of warm air blanket and warm fluids.

The patient underwent extensive escharatomies on the day of admission as a part of his initial resuscitation. He developed multiorgan failure requiring high inotropic support, renal replacement therapy and high FiO_2_. He developed hyperpyrexia (temperature >42°C) on day 11 post burn.

Relevant microbiology investigations had demonstrated no obvious focus of ongoing infection. The hyperpyrexia was resistant to conventional active cooling (bladder/gastric lavage, hemofiltration, external cooling with cooling blanket). The hyperpyrexia was associated with marked tachycardia (heart rate >150 beats/minute) with increasing oxygen demands and hypotension with escalating inotropic support. Forced core thermoregulation was commenced due to instability attributed to high core temperature.

The Thermogard XP^® ^was inserted in the femoral vein, the target temperature was set at 37°C. Within 2 hours of initiating the IVTM, the core body temperature dropped by 3°c down to 39°C. It took a further 3 hours to stabilise at the target temperature of 37°C.

**Figure 1:**
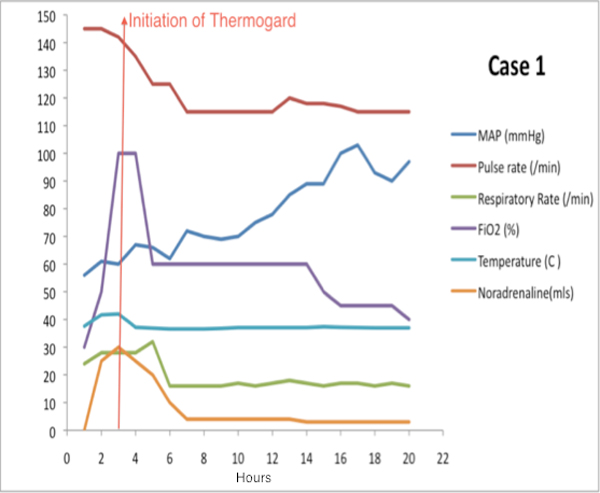
**Response in vital parameters in the first 20 hours after initiating IVTM: case 1**.

The IVTM system was used for a period of 6 days. The objective measurements of pulse rate, blood pressure, respiratory rate and urine output were seen to improve in the presence of a normothermic state (Figure 1). After a protracted and convoluted stay in the ICU, the patient was discharged to a ward after 38 days.

### Case 2

A 34-year-old female, a known intravenous drug abuser, admitted to burns ICU following an attempt of deliberate self-harm. The patient, heavily drunk, allegedly grasped a high-voltage (400 kV) live wire on a pylon and was found 15 feet away; she had sustained polytrauma requiring splenectomy for splenic rupture and chest drains for pneumothoraces. She had suffered deep thermal burns involving 27% TBSA.

**Figure 2:**
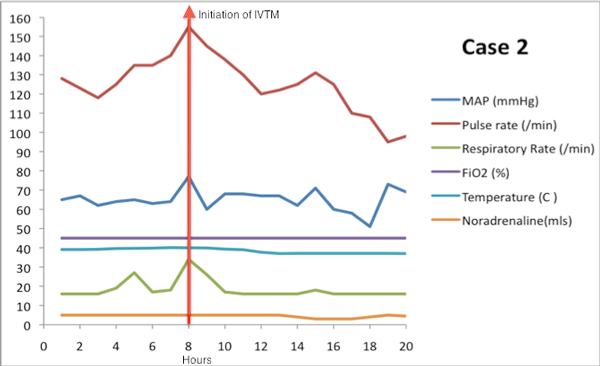
**Response in vital parameters in the first 20 hours after initiating IVTM: case 2**.

She had escharotomies on her both upper extremities and chest within hours of admission. Patient subsequently developed rhabdomyolysis, myoglobinuria and renal failure. The patient remained ventilated, requiring ionotropic support and haemofiltration. She remained hyperpyrexial from day 1 post burn; however, the temperature crept above 41°C on day 5 post burn. The temperature was persistently high despite appropriate broad-spectrum antibiotics, antifungal therapy and conventional methods of cooling. Physiological instability led to the addition of forced core thermoregulation on day 8. The target temperature of 37.5°C was achieved over a period of 4 to 8 hours with resultant improvement in pulse and respiratory rates and reduced inotrope levels (Figure 2). The forced core thermoregulation was held periodically to assess the relapse of hyperthermia and was reinstated as required.

## Discussion

Thermoregulatory failure leading to elevation of core body temperature more than 37.5°C (37.5 to 38.3°C) is hyperthermia. The core body temperature is managed in a tight range by the balance of heat production and heat loss.

The detrimental effects of hyperthermia on a patient in intensive care cannot be overstated. Hyperthermia in adults with major burns is not uncommon; however, the extent of the problem is not known. From other cohorts like patients with brain injury, it is well known that hyperthermia is an independent predictor of increased length of stay and poor outcome in the ICU [4]. The impact of hyperthermia on various organ systems and mortality depends on the degree of temperature elevation and rapidity of cooling to normal temperatures.

Among the various methods to instigate therapeutic cooling in intensive care, conventional methods could lead to treatment failure in as high as 60% of patients and the IVTM system is deemed most reliable to maintain a stable temperature [5].

Forced core thermoregulation using the IVTM system is effective in regulating labile body temperatures associated with severe burns [6]. The thermoregulation is achieved by circulation of saline via a ballooned catheter inserted into the central venous system, with automatic adjustment of saline temperature controlled by remote monitoring of patient temperature.

**Figure 3:**
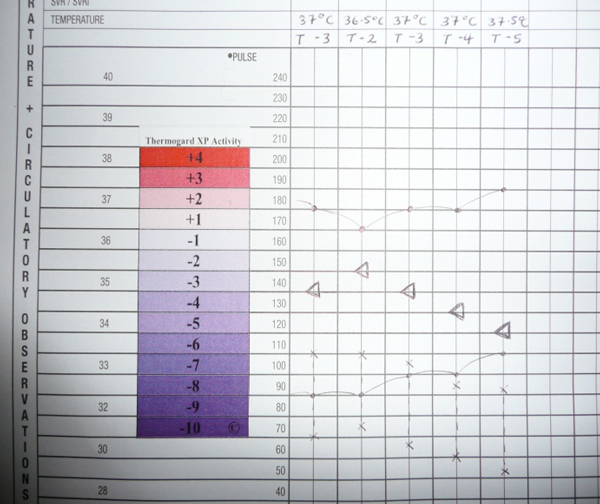
**Adhesive label that replicates the cooling/warming scale displayed on the Thermogard XP^® ^screen, allowing an arrow to be documented on the chart**.

The downsides of using IVTM systems are the need for a central venous access and complications related to it, cost factor and also use of thermoregulatory systems may mask underlying physiological changes, potentially leading to delayed or mismanagement of any precipitants of hypothermia or hyperthermia, for example sepsis. Documentation of an artificially maintained temperature on an observation chart without reference to Thermogard XP^® ^activity has implications for both immediate patient management and retrospective review of observation charts for audit or medico-legal purposes. Currently, facilities to download data directly from the Thermogard XP^® ^electronic system are cumbersome and not readily available in most ward settings. We devised and used a qualitative method of documenting Thermoguard XP^® ^activity. Firstly, a 'T' on the observation chart to denote the presence of a Thermogard XP^® ^*in situ*. Secondly, a number assigned from a scale of +4 to -10 to represent the extent of warming or cooling, correlating with the bars displayed on the Thermogard XP^® ^screen. These two pieces of information were documented alongside the patient's core temperature every time observations are performed. We, in addition, developed an adhesive label that replicates the cooling/warming scale displayed on the Thermogard XP^® ^screen, allowing an arrow to be documented on the chart as depicted on the screen (Figure 3.) It provided a more visual representation of Thermogard XP^® ^activity and was used as an adjunct to the above numerical scale. All members of the multidisciplinary team could observe our method of documenting trends in core body temperature/Thermoguard XP^® ^activity at a glance. It is reproducible in any unit caring for critically ill burns patients.

## Conclusion

The directed response of hyperthermia in febrile and nonfebrile states has physiological merits when considering the requirements of inflammatory mediators and cells. Forced core thermoregulation has aided us in the early management of unstable intensive care patients with refractory hyperthermia.The use of an IVTM system and subsequent documentation of artificially maintained temperature can be misleading and has implications for patient management and retrospective review.
